# Differential expression of a BMP4 reporter allele in anterior fungiform versus posterior circumvallate taste buds of mice

**DOI:** 10.1186/1471-2202-11-129

**Published:** 2010-10-13

**Authors:** Ha M Nguyen, Linda A Barlow

**Affiliations:** 1 Rocky Mountain Taste and Smell Center, Department of Cell and Developmental Biology, University of Colorado Denver, School of Medicine, Aurora, Colorado 80045, USA

## Abstract

**Background:**

Bone Morphogenetic Protein 4 (BMP4) is a diffusible factor which regulates embryonic taste organ development. However, the role of BMP4 in taste buds of adult mice is unknown. We utilized transgenic mice with LacZ under the control of the BMP4 promoter to reveal the expression of BMP4 in the tongues of adult mice. Further we evaluate the pattern of BMP4 expression with that of markers of specific taste bud cell types and cell proliferation to define and compare the cell populations expressing BMP4 in anterior (fungiform papillae) and posterior (circumvallate papilla) tongue.

**Results:**

BMP4 is expressed in adult fungiform and circumvallate papillae, i.e., lingual structures composed of non-taste epithelium and taste buds. Unexpectedly, we find both differences and similarities with respect to expression of BMP4-driven ß-galactosidase. In circumvallate papillae, many fusiform cells within taste buds are BMP4-ß-gal positive. Further, a low percentage of BMP4-expressing cells within circumvallate taste buds is immunopositive for markers of each of the three differentiated taste cell types (I, II and III). BMP4-positive intragemmal cells also expressed a putative marker of immature taste cells, Sox2, and consistent with this finding, intragemmal cells expressed BMP4-ß-gal within 24 hours after their final mitosis, as determined by BrdU birthdating. By contrast, in fungiform papillae, BMP4-ß-gal positive cells are never encountered within taste buds. However, in both circumvallate and fungiform papillae, BMP4-ß-gal expressing cells are located in the perigemmal region, comprising basal and edge epithelial cells adjacent to taste buds proper. This region houses the proliferative cell population that gives rise to adult taste cells. However, perigemmal BMP4-ß-gal cells appear mitotically silent in both fungiform and circumvallate taste papillae, as we do not find evidence of their active proliferation using cell cycle immunomarkers and BrdU birthdating.

**Conclusion:**

Our data suggest that intragemmal BMP4-ß-gal cells in circumvallate papillae are immature taste cells which eventually differentiate into each of the 3 taste cell types, whereas perigemmal BMP4-ß-gal cells in both circumvallate and fungiform papillae may be slow cycling stem cells, or belong to the stem cell niche to regulate taste cell renewal from the proliferative cell population.

## Background

The mouse tongue contains three types of taste papillae: fungiform, circumvallate, and foliate. Each papilla houses one (fungiform) or many taste buds (circumvallate and foliate). In mice, each taste bud contains approximately 60-100 taste cells that are divided into 4 types: I, II, and III elongated or fusiform cells, and round, basal type IV cells. Type I cells are thought to have a glial function within the bud, and express blood group H antigen, a membrane-associated carbohydrate moiety, and GLAST, a glutamate-aspartate transporter often present in glial cells, as well as NTPdase2, a member of the family of calcium-dependent ecto-ATPases [1-3]. Recent studies suggest that type I cells may also function in salt taste transduction [4]. Type II cells are receptor cells, which transduce sweet, bitter and umami stimuli [5-8], and overlapping subsets of type II cells are immunoreactive for α-gustducin, phospholipase Cβ2 [9,10], and the inositol 1,4,5-triphosphate receptor 3 (IP_3_R3) [6]. α-gustducin-knockout mice are insensitive to bitter tastants [11], linking this particular marker to the bitter sensitive subpopulation of type II cells [12]. Type III cells transduce sour stimuli [13,14] and salty [15], and form synapses with nerve fibers [16-20]. This latter cell type expresses NCAM [21] and is serotonin immunopositive [22], as well as immunoreactive for SNAP-25 [23].

In the adult tongue, cells within taste buds undergo continual turnover; peripheral epithelial cells around taste buds are proliferative, while elongated cells and Type IV cells within taste buds are post-mitotic [24-27]. Both the intragemmal (within taste buds) basal and perigemmal (adjacent to taste buds) epithelial cell populations have each been suggested to be responsible for generating cell types I-III [26,28,29]. Birthdating studies, however, imply that perigemmal cells are the exclusive progenitors for taste buds cells, as proliferating cells are observed only around taste buds, and then appear to become post-mitotic, enter the taste bud as immature taste cells, and differentiate, as they move from the border region into the central region of taste buds [25-27,29]. For example, immature type II taste cells are born at least 2-3 days before they express specific type II cell markers [30,31].

Little is known about molecular regulation of taste cell turnover, although a number of well known signaling pathways are expressed in cells within and surrounding taste buds. For example, *Sonic hedgehog *(*Shh*) is expressed in basal cells (type IV) within taste buds, while the Shh receptor, *Patched1 *(*Ptch1*), is expressed in perigemmal epithelial cells adjacent to *Shh*-expressing cells [32,33]. *Mash1*, a transcription factor in the Notch pathway, is expressed in a subset of differentiated type III cells, and is also expressed in a subset of *Shh *expressing basal cells, suggesting a possible function for Mash1 in the transition from basal cells to type III cells [29,34]. *Prox1*, another transcription factor, has strong expression in basal cells and weak expression in elongated cells, again suggesting a role in taste cell differentiation [35]. *Sox2*, an HMG box transcription factor implicated in stem cell regulation in numerous other systems [36,37], is expressed in large numbers of circumvallate taste cells residing in the basal and middle compartment of each bud, as well as in perigemmal cells, again suggestive of a role in differentiation of immature taste cells into mature taste cells [38,39].

Bone morphogenetic proteins (BMPs) are multifunctional signaling molecules that belong to the transforming growth factor ß (TGFß) superfamily. BMPs regulate many cellular processes, including differentiation, proliferation, apoptosis, adhesion, and migration (reviewed by Chen, 2004) [40]. In the developing taste organs of embryos, BMP4 is precisely co-expressed with *Shh *in taste placodes [41]. A recent study using cultured embryonic tongue explants revealed that BMP influences formation of fungiform taste papillae [42]. In adult mice, however, the role of BMP in taste cell renewal has not been explored.

Following up on a report that BMP4 is expressed in mature taste buds [43], we have examined the pattern of BMP4-ß-gal expression in both circumvallate and fungiform taste buds with respect to taste cell type, and proliferative state. Interestingly, we find both differences and similarities in the expression pattern of BMP4-ß-gal in fungiform versus circumvallate taste buds. In fungiform papillae, BMP4-ß-gal-expressing cells are located exclusively in a small number of basal epithelial cells outside of taste buds and are never encountered inside taste buds; whereas in the circumvallate papilla, BMP4-ß-gal is expressed by perigemmal cells, as well as by immature taste cells and a subset of each of the 3 elongate taste cell types. These data suggest both conserved and divergent roles for BMP4 in these 2 papillae. Specifically we find that in both fungiform and circumvallate papillae BMP4-ß-gal marks a slowly dividing, perigemmal taste bud stem cell population and/or local signaling center, which perhaps regulates taste cell renewal, while, unique to the circumvallate papilla, BMP4-ß-gal is expressed additionally in differentiating taste cells.

## Results

### BMP4-ß-gal is expressed in epithelial and taste cells of the circumvallate papilla, but only in perigemmal epithelial cells around taste buds in fungiform papillae

To determine the expression pattern of BMP4-driven ß-galactosidase (ß-gal) in taste buds, we examined the distribution of ß-gal immunopositive or X-gal reactive cells in the tongues of BMP4^LacZ ^hemizygous mice [44]. This mouse line has been engineered so that a nuclear LacZ coding sequence replaces that of BMP4 at the native locus. Thus ß-galactosidase protein expression is a reliable reporter of native BMP4 expression [44-48], although ß-gal expression likely persists longer than BMP4 protein [49-51] (and see discussion).

**Figure 1:**
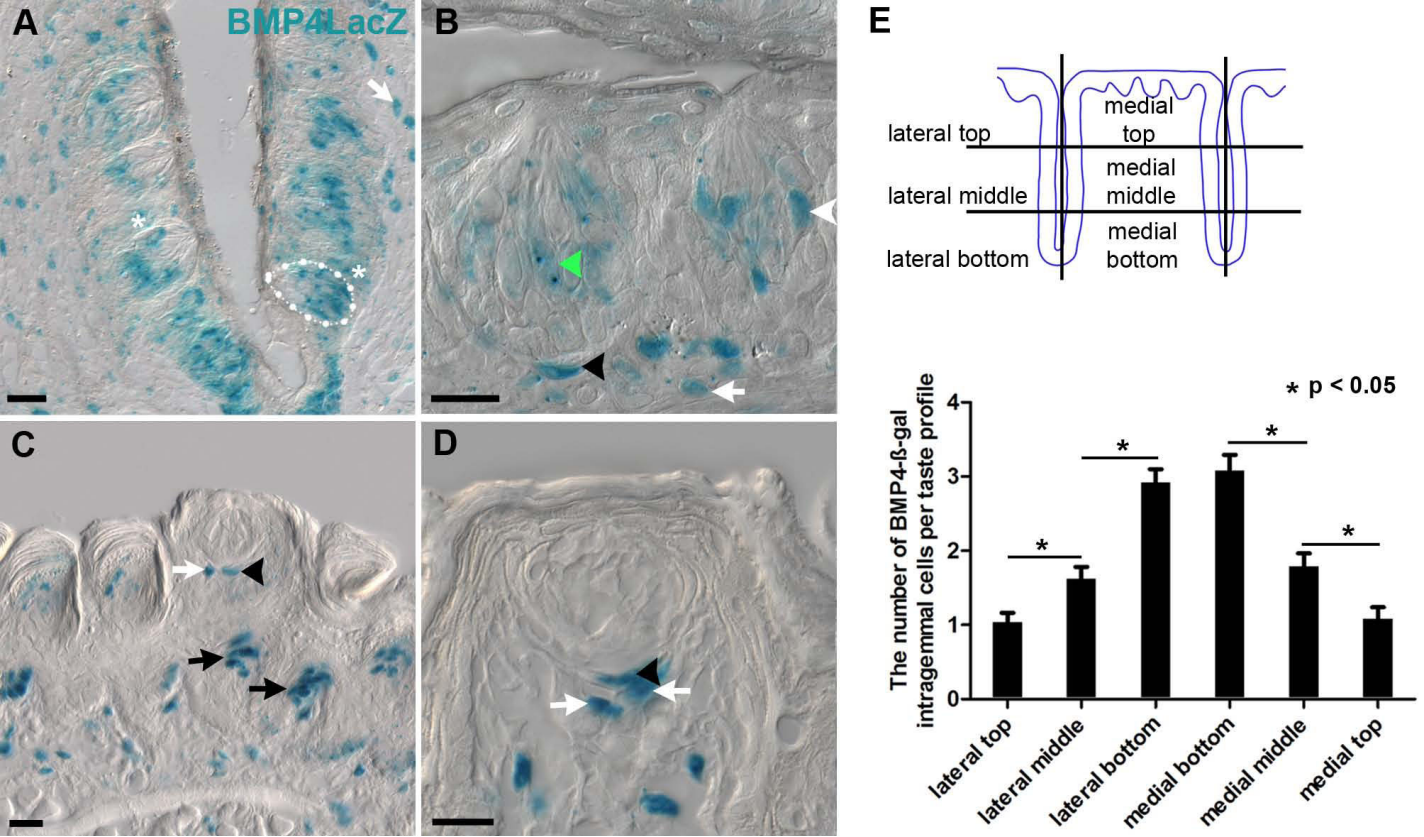
**BMP4-ß-gal is expressed in both circumvallate and fungiform papillae of adult mice**. A. In the circumvallate papilla, X-gal positive cells are evident in variable numbers within taste buds (intragemmal cells - one example outlined with white dashed line, and in papillary epithelium adjacent to taste buds (perigemmal cells, white asterisks), as well as in the subepithelial mesenchyme (white arrow). B. The heterogeneous nature of BMP4lacZ expression is evident at higher magnification. Some intragemmal fusiform cells express moderate levels of ß-galactosidase (green arrowhead), while others have higher levels (white arrowhead). Some basal epithelial cells are also strongly X-gal positive (black arrowhead), as are subepithelial lamina propria cells (white arrow). C. In fungiform papillae, BMP4-ß-gal is expressed in epithelial cells around fungiform taste buds (black arrowhead) and filiform papillae (black arrows), and is also evident in lamina propria beneath fungiform taste buds (white arrow). D. X-gal positive epithelial cells are common perigemmally in the epithelium (black arrowhead), as are positive mesenchymal cells (white arrows). E. A schematic diagram of circumvallate papillae is divided into six regions for each trench: lateral top, lateral middle, lateral bottom, medial bottom, medial middle, and medial top for each of the paired circumvallate trenches. The number of BMP4-ß-gal intragemmal cells per taste bud profile in the bottom of the papilla is significantly greater than in the middle part, and the middle part has significantly more BMP4-ß-gal cells than the top region (p < 0.01, Tukey comparison test); data indicate mean +/- standard error; n = 6 mice; at lease 50 taste buds were counted for each location. Scale bars: 20 μm.

In circumvallate papillae, BMP4-ß-gal is present in perigemmal cells adjacent to taste buds proper (Figure 1A; white asterisks, 1B; black arrowhead), as well as in intragemmal fusiform cells (Figure 1A,B; dotted outline in A shows a taste bud profile). Although we did not quantify this, we also noticed that there appeared to be different levels of transgene expression within circumvallate taste cells, with some cells expressing high levels (Figure 1B; white arrowhead), while other cells had much lower X-gal activity (Figure 1B; green arrowhead). From our first perusal of the circumvallate papilla expression pattern, it appeared that more intragemmal cells were BMP4-ß-gal positive in the deeper part of each circumvallate trench (Figure 1A). This bore true after quantitative analysis; the number of BMP4-ß-gal intragemmal cells per taste bud was significantly greater in the bottom part of papilla (Figure 1E; 378 taste buds from 6 mice, p < 0.01, ANOVA). However, we detected no significant difference in the number of BMP4-ß-gal expressing cells per bud with respect to medial versus lateral epithelium location with the circumvallate papilla.

By contrast, in the anterior tongue, BMP4-ß-gal was detected in filiform papillae (Figure 1C; black arrows) and in fungiform papillae, where perigemmal epithelial cells adjacent to taste buds displayed ß-galactosidase activity (Figure 1C,D; black arrowheads). However, ß-gal positive cells were never present within cells detected inside of taste (n = 6 animals, 50 taste buds per animal). These expression patterns were identical using either X-gal histochemical staining or anti-ß-galactosidase immunostaining. This dichotomy in BMP4-ß-gal expression with respect to papillae type suggested that: 1) BMP4-ß-gal marks a subset of perigemmal taste bud progenitor cells in both fungiform and circumvallate papillae; and 2) in the circumvallate, BMP4-ß-gal is also labeled by a subset of taste cells, perhaps those in the process of differentiating.

**Figure 2:**
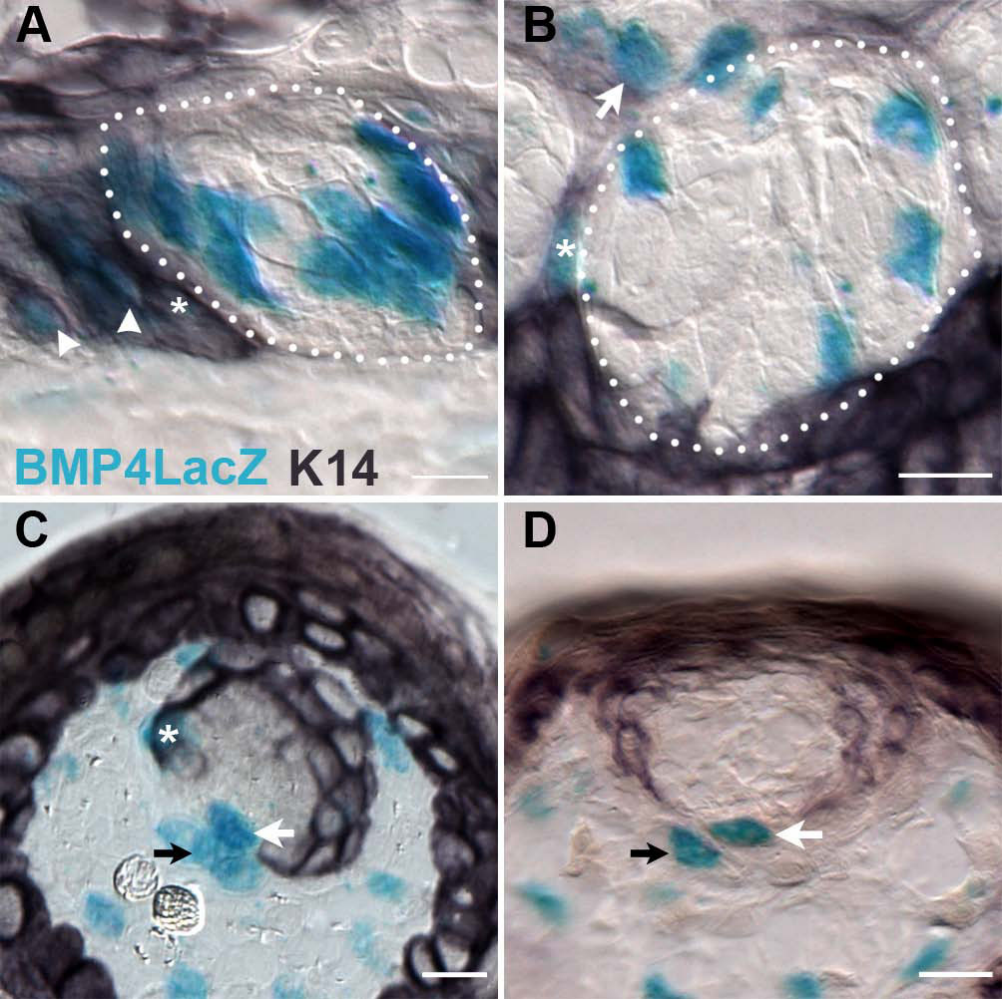
**BMP4-ß-gal is expressed in perigemmal cells of both circumvallate and fungiform papillae**. A, B. X-gal positive cells in the circumvallate papilla comprise both extragemmal (A; white arrowheads) and perigemmal (A and B; white asterisks) K14-immunopositive cells. Some perigemmal BMP4-postivie cells are not K14-IR (B, white arrow). C, D. In fungiform papillae, perigemmal BMP4-LacZ expressing epithelial cells are K14-immunonegative (C, D; white arrows), or K14-IR (C, white asterisk). Black arrows indicate X-gal positive mesenchymal cells in close apposition to X-gal positive perigemmal epithelial cells. Scale bars: 20 μm.

We also detected BMP4-ß-gal in the mesenchyme of the lamina propria beneath taste buds in both circumvallate and fungiform papillae (Figure 1, all panels; white arrows). To further define the epithelial versus mesenchymal identity of the BMP4 cells in both circumvallate and fungiform papillae, we performed double labeling for BMP4-ß-gal and cytokeratin 14-IR (K14), which marks basal keratinocytes of oral epithelia [52]. We observed several patterns of labeling in both taste papilla types: (1) In the same section, we found BMP4-ß-gal extragemmal cells (epithelial cells in papillae with at least 1 cell distant from taste buds) outside taste buds and perigemmal cells (immediately adjacent to taste buds) were double-labeled with K14 (Figure 2A; extragemmal cells - white arrowhead; Figure 2A,B,C; perigemmal cells - white asterisks); (2) K14 immunonegative BMP4-expressing perigemmal cells located near K14-immunopositive cells in the epithelium (Figure 2B,C,D; white arrows); and (3) Lamina propria (black arrows) and epithelial (white arrows) BMP4-ß-gal and K14-immunonegative cells immediately adjacent to one another (Figure 2C,D).

### BMP4-ß-gal is expressed in a small percentage of all three differentiated taste cell types in circumvallate papillae

**Figure 3:**
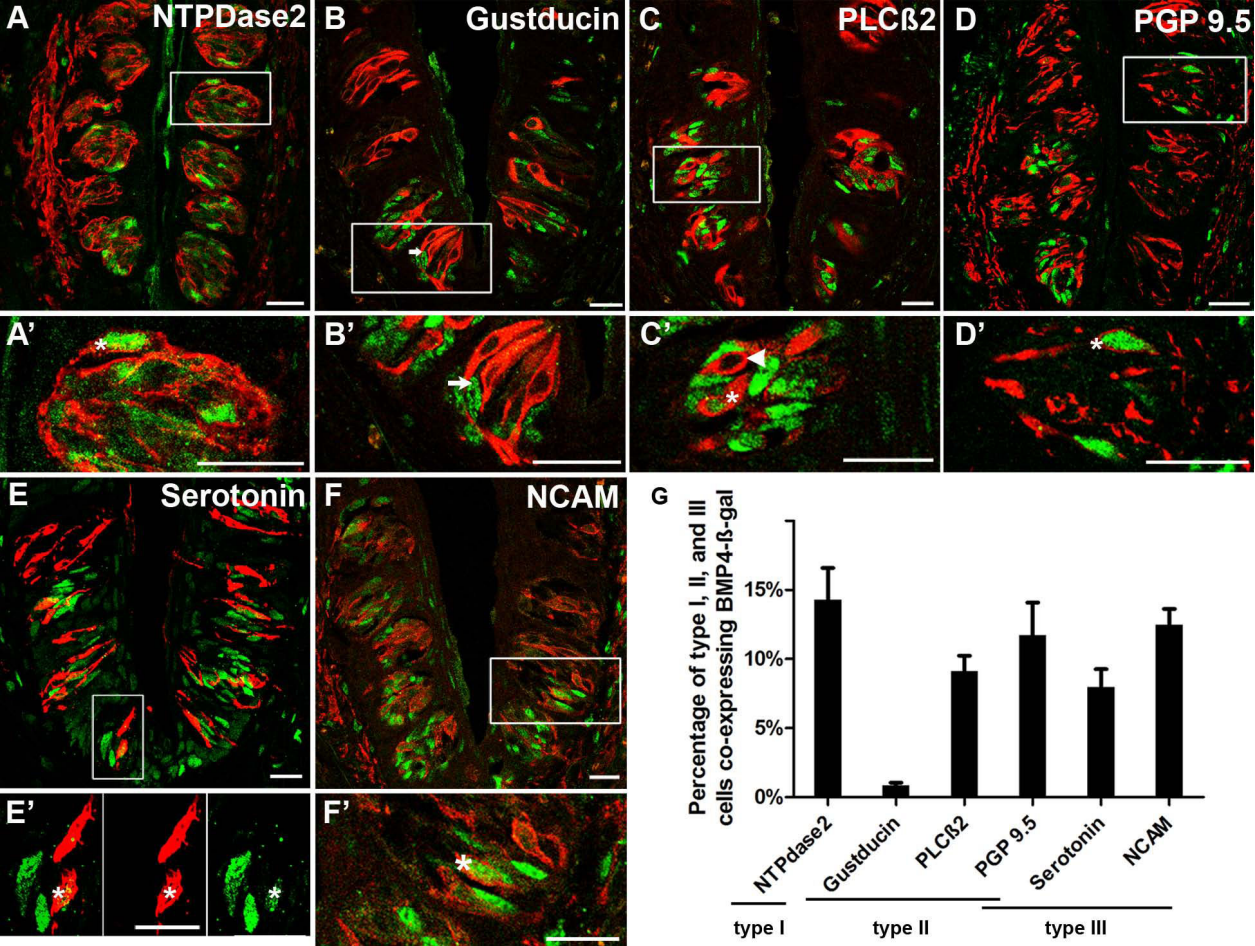
**BMP4-ß-gal is expressed in a subset of each differentiated taste cell type in circumvallate papillae**. Double labeling of BMP4-ß-gal-IR (green nuclei) with differentiated taste cell immunomarkers (red) in circumvallate taste buds. A, A'. Double immunostaining for ß-gal and NTPDase2 (type I cells) reveals double labeled cells (arrowhead in A'). B, B'. In a double immunostained section for BMP4-ß-gal and Gustducin-IR (Type II cells), an arrow indicates one of several cells immunopositive for BMP4-ß-gal but not for gustducin. C, C'. Double staining for BMP4-ß-gal with PLCß2 (type II cells) showing both double labeled (white arrowhead) and singly labeled type II cells (white asterisk). D, D'. Double staining for BMP4-ß-gal with PGP9.5 (subset of type II and III cells) also reveals a double labeled cell (white arrowhead), as well as cells expressing one or the other immunomarker. E, E'. Double staining for BMP4-ß-gal with serotonin-IR (Type III cells) again shows single and double (white arrowhead) labeled taste cells. The boxed area shown in E, is shown in E' as a high magnification view of the merged and channels to better demonstrate double labeling F, F'. Double immunostaining for BMP4-ß-gal with NCAM-IR (type III cells) reveals double (arrowhead) and singly labeled taste cells. A',B',C',D',E', and F' are higher magnification of white boxes in A,B,C,D,E, and F respectively. Scale bars: 20 μm. G: Percentages of type I, II and III taste cells co-expressing BMP4-ß-gal in circumvallate taste buds (mean +/- standard error of the mean, n = 3 mice; 100 to 150 taste buds were counted for each taste cell type marker).

To further characterize the expression of BMP4-ß-gal in circumvallate taste bud cells, we performed double immunofluorescence for ß-gal and mature taste cell markers. Double staining with antisera against known taste cell markers revealed a small number of all 3 fusiform taste cell types (I, II, III) were also immunoreactive for BMP4-driven ß-gal (Figure 3). Using anti-NTPDase2 to detect type I cells [3], we observed that 14.3% of NTPDase2- immunoreactive (IR) cells were also ß-gal-IR (Figure 3A,A',G; > 50 taste buds per animal, n = 3 mice). To detect the extent of BMP4-ß-gal expression in type II cells, we employed anti-gustducin or anti-PLCß2 antisera (Figure 3B,B',C,C'). Very few double-labeled gustducin-IR cells were encountered, while 9.1% of PLCß2-IR cells expressed ß-gal (Figure 3G). NCAM and serotonin antisera were used to assess BMP4-ß-gal expression in type III cells (Figure 3E,E',F,F'), and 8% of serotonin-IR cells and 12.5% of NCAM-IR cells were ß-gal-immunopositive (Figure 3G). Finally, PGP9.5 antiserum recognizes a subset of both type II and III cells, which are not immunoreactive for gustducin or serotonin, respectively [17]. Double immunolabeling for ß-gal and PGP9.5 revealed that 11.7% of PGP9.5-IR expressing cells also were ß-gal-IR (Figure 3D,D',G).

In contrast to BMP4-ß-gal expression within circumvallate taste buds and as expected from our initial analysis of Xgal staining, fungiform taste buds were virtually devoid of BMP4-ß-gal, and thus were not co-labeled with markers of differentiated taste cells (data not shown). In the anterior tongue, intragemmal BMP4-ß-gal was never detected in fungiform papillae. Interestingly, however, there were rare cases of ß-gal expression in the middle and posterior fungiform taste buds (data not shown). The type(s) of taste cells expressing BMP4-ß-gal was not explored further, as their incidence was infrequent.

### Co-expression of BMP4-ß-gal and Sox2

**Figure 4:**
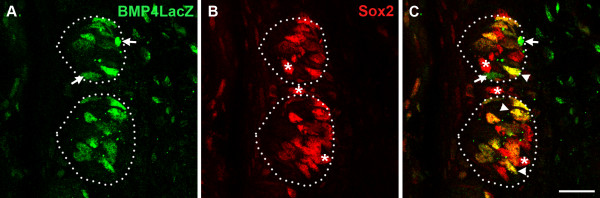
**BMP4-ß-gal is expressed in taste cells expressing Sox2 in circumvallate papillae**. BMP4-ß-gal-IR (A, green) is expressed in fusiform cells of circumvallate taste buds, and a large number of them are Sox2-IR (B, red). In C, white arrowheads indicate double-labeled cells. In B and C, white asterisks indicate intragemmal and perigemmal cells that are Sox2-IR only, while in A and C, white arrows point to BMP4-ß-gal-IR cells that are not Sox2-IR. Scale bar: 20 μm.

Because BMP4-ß-gal was expressed in only a small percentage of mature taste cells within the circumvallate papilla, we tested if BMP4-ß-gal co-localized with Sox2, a putative marker of immature taste cells [38]. Sox2 is involved in embryonic taste bud formation [39], and has been suggested to play a role in the differentiation of all 3 taste cell types in circumvallate taste buds [38]. Consistent with the hypothesis that BMP4 is expressed in immature taste cells, we found most, but not all, Sox2-IR taste cells were also BMP4- ß-gal-IR (Figure 4C; yellow cells indicated with white arrowheads), although many Sox2-IR were not double labeled (Figure 4B,C, red cells, white asterisks). Similarly, while most BMP4-ß-gal-IR cells were immunoreactive for Sox2, singly labeled BMP4 cells were readily detected (Figure 4A,C; green cells, white arrows).

### Birthdating of BMP4-ß-gal -expressing taste cells within circumvallate taste buds

**Figure 5:**
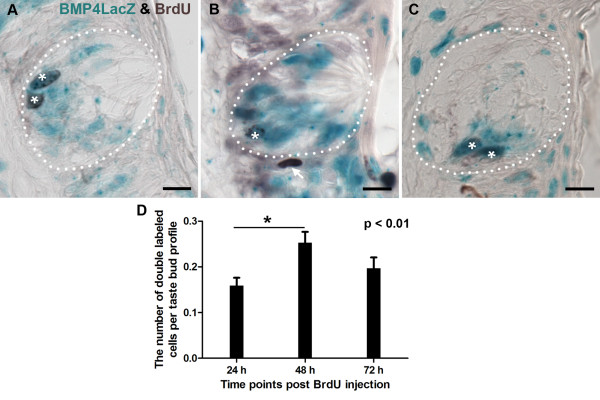
**In circumvallate taste buds, intragemmal cells express BMP4-ß-gal within 24 hours of cell birth**. Co-expression of BMP4-ß-gal (Xgal; blue) and BrdU (dark brown) was detected beginning at 24 hours after BrdU injection (A), and was still observed at 48 hours (B), and 72 hours (C). White asterisks indicate double-labeled intragemmal cells. An arrow in B points to a BrdU immunopositive perigemmal cell that does not express BMP4-ß-gal. D. The mean number of intragemmal cells labeled for both BrdU and BMP4-ß-gal is plotted with respect to time post-BrdU injection. The incidence of double labeled cells per bud profile peaks at 48 hrs. Taste buds were tallied throughout the circumvallate papilla, and only taste buds with a clear taste pore were included. (Mean +/- standard error, n = 3 mice for each time point, At least 200 taste buds were counted for each time point). Scale bars: 10 μm.

We hypothesized from our analysis of expression of Sox2-IR and of immunomarkers of differentiated taste cells, that the BMP4-ß-gal immunopositive intragemmal cells in circumvallate taste buds may represent an early phase of taste cell differentiation. To test the idea that immature taste cells express BMP4-ß-gal as they undergo differentiation, mice were injected with BrdU, and then sacrificed at 24 hour intervals, up to 72 hours post-injection, encompassing the time frame when type II and type III taste cells are known to begin differentiation [31,53]. At 24 hours post-injection, some BMP4-ß-gal cells inside taste buds were BrdU-IR (mean +/- SEM: 0.16 +/- 0.02 double-labeled cells per taste bud; Figure 5A, white asterisks, D), consistent with the idea BMP4-ß-gal is expressed by newly generated taste cells. At 48 and 72 hours post-injection, double-labeled cells were also observed in the basal region of taste buds (Figure 5B,C; white asterisks). Specifically at these 2 later time points, the number of co-labeled cells was 0.25 +/- 0.02 at 48 hours post-injection, and 0.20 +/- 0.02 at 72 hours post-BrdU injection (Figure 5D). The number of double labeled taste bud cells showed a statistically significant increase at 48 hours post-injection compared with 24 hours post-injection (p < 0.01, Tukey comparison test), but not with 72 hours post-injection. These data suggest that BMP4 is expressed in immature taste cells as they enter the taste bud, and this expression may mark a transition stage as immature cells differentiate into mature taste cells.

### The relationship between proliferating epithelial cells and BMP4-ß-gal perigemmal cells

**Figure 6:**
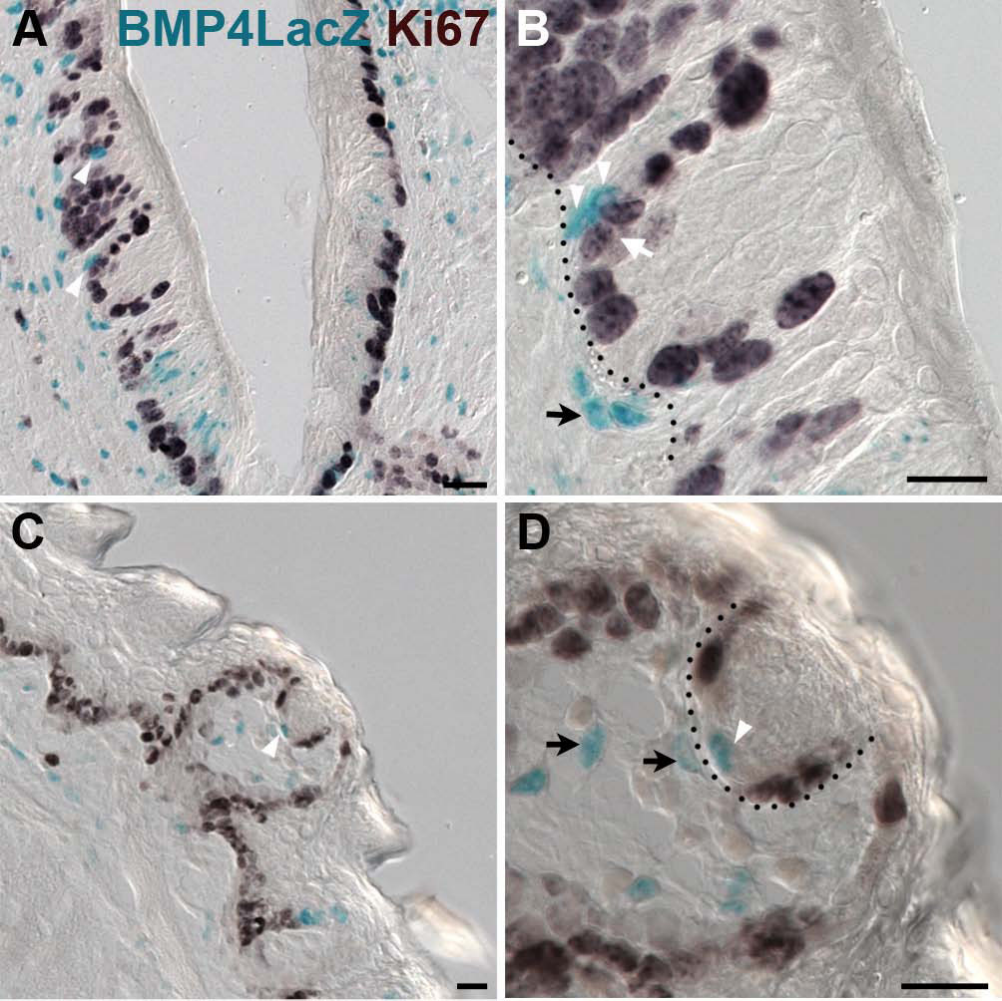
**BMP4-ß-gal perigemmal cells in fungiform and circumvallate papillae are not actively cycling**. BMP4-ß-gal expression (X-gal reaction; blue) and Ki-67 immunostaining (dark brown) in circumvallate (A,B) and fungiform papillae (C,D) reveals that cycling cells surround taste buds basolaterally, but that BMP4-ß-gal positive epithelial cells do not reside in this mitotic domain. In circumvallate papillae (A,B) and fungiform papillae (C,D), white arrowheads indicate BMP4-X-gal positive cells in the epithelium that are not Ki-67 positive, although X-gal positive epithelial cells are located near perigemmal Ki-67 immunopositive cells (e.g. white arrow in B). Black arrows indicate BMP4 expressing cells in adjacent mesenchyme, in close proximity to taste buds and BMP4-Xgal positive perigemmal epithelial cells. In B and D, the black dotted line delineates the basement membrane separating the epithelium and mesenchyme. Scale bars: 20 μm.

**Figure 7:**
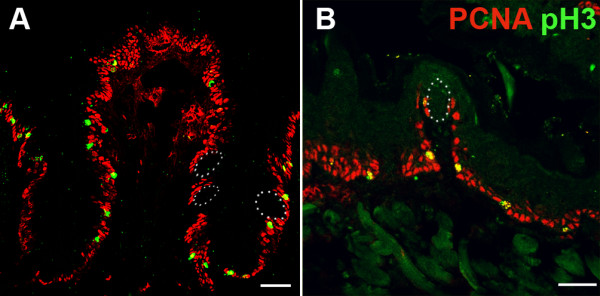
**Proliferating and mitosing cells are basal epithelial and perigemmal cells in both fungiform and circumvallate taste papillae**. Proliferating PCNA-IR cells (red) and mitosing pH3-IR cells (green) in the circumvallate (A) and fungiform papillae (B) are located perigemmally, and not inside taste buds (white dashed outlines). Scale bars: 50 μm.

In circumvallate and fungiform papillae, BMP4-ß-gal expressing perigemmal epithelial cells are also found adjacent to taste buds, suggesting that these cells may be involved in the continual renewal of adult taste cells. To address this issue, we assessed the distribution of actively cycling cells in circumvallate and fungiform taste papillae, with the expectation that a few stem cells and all transit amplifying cells would be mitotically active. We employed 4 immunomarkers to identify dividing cells: 1) proliferating cell nuclear antigen (PCNA) present in all phases of cell cycle except early G1 [54-56]; 2) Ki-67, which detects all phases in actively cycling cells, i.e., G1, S, G2, and M [57]; 3) BrdU, which is incorporated into DNA during S phase [58]; and 4) Phospho-Histone 3 (pH3) which marks cells in M phase (reviewed by Norbury and Nurse, 1992) [59]. The vast majority of basal epithelial cells of both the fungiform and circumvallate taste papillae were actively cycling, as evidenced by broad Ki-67 (Figure 6) and PCNA-IR (Figure 7 - red), while a small percentage of cells were mitosing and were pH3-IR (Figure 7 - green). In both circumvallate and fungiform papillae, a significant number of perigemmal cells were Ki-67-immunopositive or PCNA-IR (Figure 7). However, cells expressing proteins indicative of actively cycling cells were never encountered within taste buds, only in adjacent taste epithelial cells, consistent with the model that epithelial cells around taste buds are proliferative and give rise to immature, postmitotic taste cells, which then enter the taste buds [24,27,52].

Because BMP4-ß-gal perigemmal cells appeared to reside in the proliferative regions of each of the taste papillae, we investigated whether BMP4-ß-gal perigemmal cells adjacent to taste buds were actively cycling. Using double-staining for X-gal histochemistry with Ki-67 immunocytochemistry, we found that BMP4-ß-gal perigemmal cells were never Ki-67-immunopositive (see figure 6, BMP4-ß-gal blue nuclei - white arrowheads vs. cycling Ki-67-IR black nuclei - white arrow in B; n = 3 mice for both circumvallate and fungiform papillae), indicating that BMP4 expressing cells are not mitotically active.

### Characterization of the timing of cell renewal in fungiform vs. circumvallate taste buds

**Figure 8:**
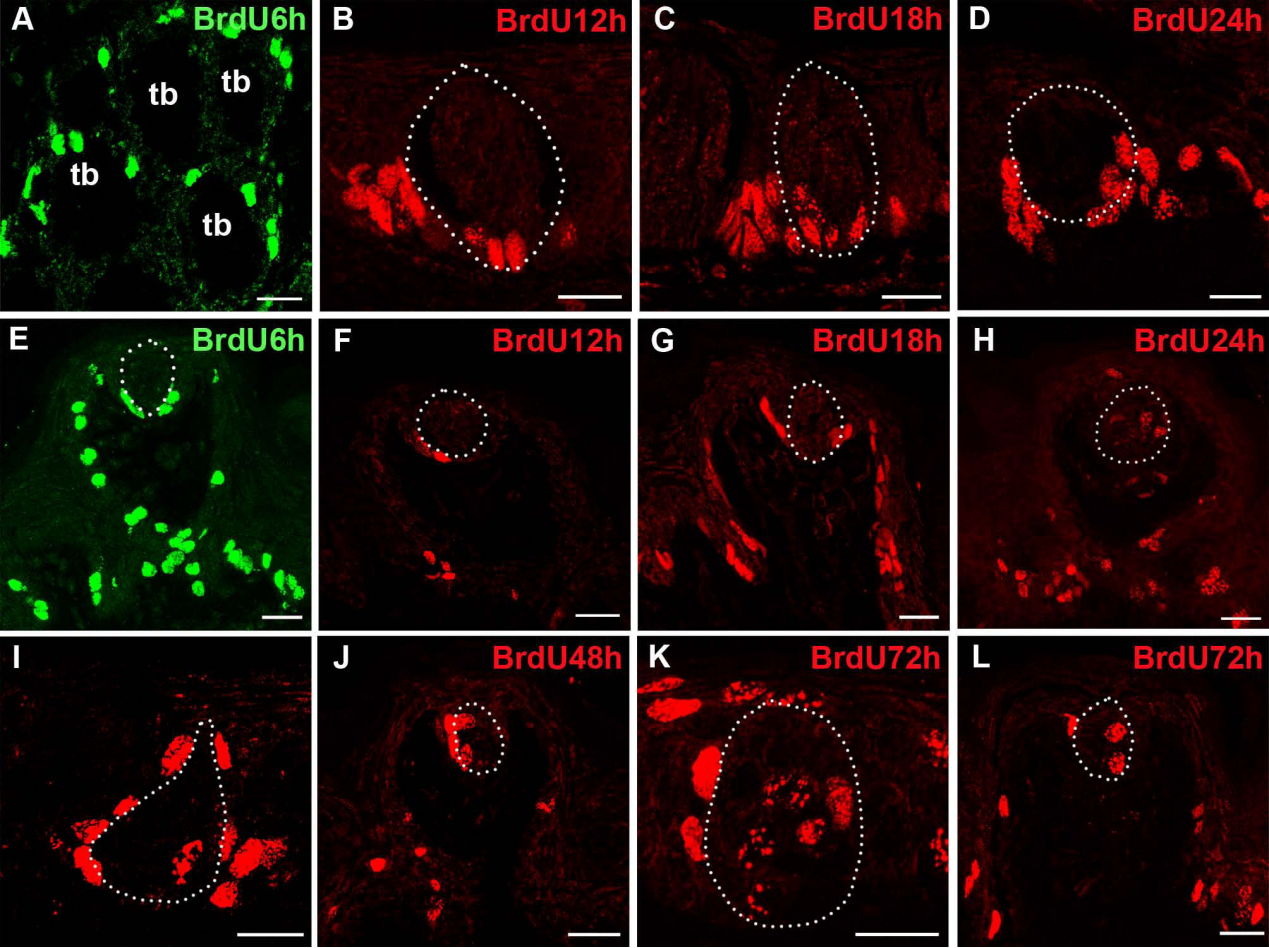
**Newly born taste cells enter taste buds in both circumvallate and fungiform papillae within 12-18 hours of birth**. A,E. BrdU-IR cells are present in the basal epithelium and perigemmaly, around taste buds, at 6 hours post-injection in circumvallate papillae (A, transverse section) and fungiform papillae (E); tb = taste bud. B,F. At 12 hours post-injection, BrdU-IR cells are evident around taste buds, and some BrdU-IR cells are found inside taste buds in the circumvallate papilla (B circumvallate; F fungiform). C,G. In both circumvallate and fungiform papillae, BrdU-IR cells are present inside taste buds at 18 hours post-injection (C circumvallate; G fungiform). D,H. BrdU-IR cells reside in the basal compartment and along the inner limits of taste buds by 24 hours (D circumvallate; H fungiform). I,J,K,L. BrdU-IR cells are detected in more central regions of taste buds at 48 hours (I circumvallate; J fungiform) and 72 hours (K circumvallate; L fungiform), but are also still detected in the perigemmal zone (white arrows). Scale bars: 20 μm.

Because BMP4-ß-gal intragemmal cells are absent in fungiform taste buds, yet are present in circumvallate buds, we wondered if this difference reflected differences in the timing of taste cell renewal between the papillae; are circumvallate taste cells generated more rapidly from the proliferative pool, and thus persistent BMP4-ß-gal expression inside taste buds is an artifact of ß-galactosidase perdurance? To assess if the rate of cell movement into taste buds differs between circumvallate and fungiform taste buds, mice were injected with BrdU, and then harvested at distinct times after injection. Initially, 6 hours after BrdU injection, after cells in S phase had incorporated the labeled nucleotide, all labeled cells are found outside of both circumvallate and fungiform taste buds, in the basal papilla epithelium adjacent to buds, as well as elsewhere in taste papillae (Figure 8A,E). BrdU-labeled cells were never detected within taste buds at this early time point. However, at 12 hrs post-BrdU injection, some BrdU-IR cells were observed inside circumvallate taste buds, but not inside fungiform taste buds (Figure 8B,F). By 18 hours post-injection, however, BrdU-IR cells were detected in both circumvallate and fungiform taste buds (Figure 8C,G), indicating that immature taste cells enter taste buds between 12 and 18 hours after their last division. By 24 hours, numerous BrdU-IR cells were evident within taste buds in both papillae (Figure 8D,H), consistent with the reports of others that have detected BrdU-IR cells inside taste buds at 12-24 hours post-injection [26,29]. Numerous BrdU-IR intragemmal taste cells were present 48 and 72 hours post injection (Figure 8I-L). Importantly, despite the differences in BMP4-ß-gal expression in fungiform versus circumvallate taste buds (see Figures 1 &3), we did not detect any significant difference in cell cycle kinetics for taste cell genesis between these 2 different taste fields (Table 1; p > 0.05, t-tests between the 2 papillae at each time point). Thus, the rate at which new cells are contributed does not differ between circumvallate and fungiform taste buds.

**Table 1:** New cell entry is comparable in circumvallate and fungiform taste buds

Time post-BrdU injection	Number of circumvallate taste bud profiles (number of mice)	Number of circumvallte taste bud profiles with BrdU-IR cells	Mean number of BrdU-IR cells per taste bud profile +/- SEM (for all cvp taste buds)	Number of fungiform taste bud profiles (number of mice)	Number of fungiform taste bud profiles with BrdU-IR cells	Mean number of BrdU-IR cells per taste bud profile +/- SEM (for all ffp taste buds)
24 h	37(3)	225	0.72 +/- 0.04	52 (3)	28	0.66 +/- 0.1

48 h	314(3)	230	0.89 +/- 0.05	52 (3)	30	0.67 +/- 0.1

72 h	245 (3)	139	0.66 +/- 0.09	61 (3)	38	0.66 +/- 0.09

The number of BrdU-IR cells inside taste buds per taste bud profile at 24, 48, and 72 hours post-injection (mean +/- standard error, n = 3 mice for each time point). There is no statistical difference between the number of new cells at any time point between the circumvallate and fungiform taste buds (p > 0.05 by t test for each time point post BrdU injection).

## Discussion

BMP4 regulates embryonic development of taste papillae [41,42], but its expression and role in adult taste buds has not been investigated. We show here that BMP4, via a genetically engineered LacZ reporter mouse line, is expressed in a subset of cells associated with mature taste buds, but that the pattern of expression differs between the circumvallate and fungiform taste papillae. In the circumvallate, BMP4-ß-gal is expressed within taste buds, by a subset of cells of each taste cell type, as well as by likely immature taste cells. Perigemmal epithelial cells within the circumvallate trenches are also BMP4-ß-gal positive. By contrast, BMP4-ß-gal intragemmal cells are absent from fungiform taste buds; instead, only a small number of perigemmal epithelial cells are BMP4-ß-gal positive. These differing expression patterns suggest that BMP4 expression marks the same perigemmal cell population in both fungiform and circumvallate taste epithelia, but hints at an additional role for this gene product in within taste buds of the circumvallate papilla.

Throughout our study we have equated BMP4-ß-gal expression with that of BMP4 protein. This reporter allele comprises a lacZ knockin to the BMP4 coding region and regulation of transcription of this locus should thus be unaltered. Further, ß-galactosidase expression in this mouse line has been shown repeatedly and in a variety of tissues and developmental stages to accurately reflect *BMP4 *mRNA expression [44,45,48], including faithfully replicating *BMP4 *mRNA expression in the developing taste bud progenitor cells of mouse embryos [41,60]. Nonetheless, we were unable to confirm this concordance in adult mouse taste epithelium. We used 3 different *BMP4 *antisense probes and were never able to detect signal above background in the lingual epithelium, even though one of these probes worked well in our positive control tissue, i.e., hair follicles (data not shown). One explanation is that *BMP4 *mRNA is expressed at very low levels in adult taste tissue, and is undetectable via in situ hybridization. Given the persistence of ß-gal protein, we suspect that the BMP4LacZ line reports subtle, perhaps more short-lived, BMP4 protein expression patterns that would otherwise be missed. Alternatively, albeit less likely, *BMP4 *mRNA may be specifically degraded in taste epithelium resulting in the absence of protein, while *LacZ *transcripts escape this suppression due to differences in mRNA structure. However, given the documented conformity of *BMP4 *mRNA expression patterns with those of ß-galactosidase expression in this knockin line, we are reasonably confident that the reporter reveals BMP4 expression in adult taste epithelium.

One explanation for the differences in expression patterns and potential roles of BMP4 between circumvallate and fungiform papillae may be due to differences in their embryonic origins [61]. Although in all vertebrate, taste buds arise from local epithelium [62,63], fungiform and circumvallate taste buds may derive from epithelia of different origins. In rats, keratin 20-IR is distributed in the posterior third of the tongue, suggesting that this region, including circumvallate papillae, derives from endoderm [61], while the anterior two-thirds of the tongue, in which fungiform papillae reside, is derived from ectoderm (keratin 20-immunonegative) [61]. We have observed rare BMP4-ß-gal expressing cells inside fungiform taste buds, but only in the most posterior fungiform papillae (data not shown), which may possess this expression pattern due to an endodermal origin. These ideas remain to be tested until we obtain a clear understanding of the contributions of endoderm and ectoderm to the epithelium lining the oral cavity.

In fungiform and circumvallate taste buds, BMP4-ß-gal is expressed by a relatively small number of perigemmal epithelial cells located adjacent to taste buds, in the same general location as the progenitor population for taste cells. Stone [28], using X-inactivation mosaic mice, reported that multiple progenitors give rise to individual taste buds, and these progenitors are assumed to comprise the basal cells located at the base of taste buds, and/or perigemmal cells situated adjacent to taste buds proper [24-26]. Most recently, Okubo [52] have shown that cells within taste buds arise from K14-expressing basal keratinocytes, which sit along the basement membrane of the lingual epithelium, including that of taste papillae. Taste cells within buds do not express K14, but rather express cytokeratin 8 [52,64,65]. In our studies, while BMP4-ß-gal perigemmal cells are located in a position consistent with that of proposed progenitors and many of BMP4 cells were also K14 positive, we could find no evidence that these cells are actively dividing, via both BrdU birthdating and immunostaining for known markers of proliferation. However, it is possible that these BMP4-ß-gal cells divide so infrequently that we simply did not examine enough taste buds from enough mice at a larger range of times, although we did assay 12 mice ranging in age from 2 months to 6 months, and in no case encountered a single BMP4-ß-gal expressing cell that was in any state of proliferation. Intriguingly, another set of BMP4-ß-gal cells in the taste epithelium does not express K14; thus, while these singly labeled cells do reside within the perigemmal taste epithelium, they are not within the progenitor pool identified by Okubo and colleagues [52]. In conclusion, our expression data indicate that the perigemmal BMP4-ß-gal cells in both the circumvallate and fungiform papillae are a heterogeneous epithelial population potentially comprising: (i) a slowly cycling taste bud stem cell population (the K14-immunopositive, BMP4-ß-gal expressing cells); and (ii) a niche population or a signaling center for taste bud stem cells in combination perhaps with nearby BMP4-ß-gal cells of the lamina propria, which together may regulate taste cell genesis from taste bud stem cells.

### In circumvallate taste buds, BMP4 is expressed in early differentiating taste cells

Our birthdating analysis with BrdU is consistent with the model that taste cells are born outside of taste buds proper, and then, within a day, move into the taste bud at the margins, and ultimately come to occupy the taste bud core [24,26]. BrdU is incorporated into dividing cells outside taste buds within 1-6 hours after injection, but BrdU-IR cells are not evident in taste buds until 12-18 hours, mainly in the basal compartment. At later time points, as has been described by others, we find labeled taste cells become more centrally located, progressing toward the taste bud core within 24-72 hours post BrdU injection [27,33,65,66]. This timeframe is consistent with reports where taste cells typically express markers of differentiation by 2.5-3.5 days after birth. For example, expression of BrdU in gustducin-IR cells is first detected 2.5 days after injection [30,31].

To test the idea that BMP4 marks immature taste cells in the circumvallate papilla, we labeled newly dividing cells with BrdU and followed these cells for 3 days to assess when BMP4-ß-gal expressing fusiform cells are born. As type II cells differentiate at 2.5-3 days after birth, we predicted that, if BMP4 marks a transitional stage from transit amplifying cells to mature taste cells, we would detect BMP4-ß-gal and BrdU co-labeled cells within 2.5 days of BrdU injection. In fact, BrdU is detected in BMP4-expressing fusiform cells beginning at 24 hours post-injection, suggesting that BMP4-expressing fusiform cells are indeed immature taste cells. It is possible, however, that ß-galactosidase in taste buds persists longer than native BMP4 protein would, which in vitro, is known to decay within 6 hours [67], while ß-galactosidase has a much longer half-life (approximately 13 hours) [44,49-51]. Thus, BMP4-ß-gal inside taste buds might not accurately reflect BMP4 expression, but rather would represent residual ß-galactosidase that was intially driven by the BMP4 promoter in cells outside of taste buds. If this were the case, the number of BMP4-ß-gal and BrdU-IR double labeled cells inside taste buds should peak within 24 hours after BrdU injection, as the half-life of ß-gal is 13 hours [44,49-51]. However, we find that the number of BrdU and X-gal double positive cells inside taste buds peaks at 48 hours post-injection, well after the expected decay of ß-gal, indicating that BMP4-ß-gal inside taste buds must instead be due to active transcription and translation of lacZ under the BMP4 promoter within newly generated taste cells in the circumvallate papilla.

There are at least 3 models for cell lineage relationships in taste buds: (i) Taste cells derive from a single lineage, progressing from basal cells to type I, then to type III, and finally to type II cells during maturation [26]; (ii) Taste buds are composed of at least two cell lineages, each of which produces a subset of differentiated taste cell types [28,29,68]; or (iii) all 3 taste cell types arise via distinct lineages [29]. To determine if BMP4 expression is limited to one cell type, and potentially marks one or a subset of taste cell lineages, we examined which cell types within circumvallate taste buds expressed BMP4-ß-gal. We found that a small percentage of each taste cell type (I, II, and III) co-express BMP4-ß-gal, suggesting that intragemmal BMP4 is expressed by differentiating taste cells regardless of cell type. Except for the subset of type II cells immunopositive for gustducin, the percentages of double labeled BMP4-ß-gal and type I, II, III cells (NTPDase2, PLCß2-IR, PGP9.5-IR, serotonin-IR, NCAM-IR) are not statistically different (Tukey test; p > 0.05), tending to support the hypothesis that BMP4 is broadly expressed by differentiating taste cells, regardless of cell type. However, this may not be the case for gustducin-IR cells, which are the subset of type II cells within the circumvallate that transduce bitter taste [11,12]. As very few double-labeled gustducin-IR cells were detected, it may be that BMP4 is not involved in differentiation of the bitter-sensing cell lineage. The fact that the number of BMP4-ß-gal-expressing cells per taste bud is higher at the bottom portion of the circumvallate trenches suggests the possibility of more rapid turnover of cells in taste buds in the deeper regions of the papilla epithelium, but this idea remains to be tested.

To investigate further our hypothesis that BMP4-expressing cells within buds are immature taste cells, we assessed the expression of a proposed marker for immature taste cells, Sox2 [38], and compared this pattern to that of BMP4-ß-gal. High levels of Sox2 expression have been found in the progenitor cells that are committed to taste cell lineage [52]. We, too, found that in circumvallate taste buds, a large number of BMP4-ß-gal intragemmal cells are co-immunoreactive for Sox2, providing another piece of evidence that BMP4-ß-gal marks immature taste cells.

### What is the nature of the mitotically quiescent BMP4-ß-gal expressing epithelial cells in taste papillae?

It has been proposed that basal and/or perigemmal cells adjacent to taste buds comprise the taste progenitor population, responsible for continual generation of adult taste cells [26,28,29,69,70]. As in generalized epithelium, taste bud stem cells are thought to undergo asymmetric division to produce a progenitor daughter that goes through transit amplifying divisions to produce a number of immature taste cells [52,71]. Thus, we hypothesized initially that the BMP4-ß-gal cells were stem cells and/or transit amplifying cells of the taste bud lineage. If so, these cells should express markers of actively cycling cells, including Ki-67 and BrdU incorporation during S phase DNA synthesis. However, double labeling with Ki-67, which labels mitotically active cells in all phases of the cell cycle [57], showed that these BMP4-ß-gal expressing cells were not actively cycling. We further confirmed this lack of mitotic activity with additional markers of cell proliferation (PCNA and pH3), and in short duration birthdating studies employing BrdU. In sum, these data indicate that BMP4-ß-gal expressing cells are not rapidly dividing transit amplifying cells.

That BMP4-expressing cells found outside of taste buds are not mitotically active suggests at least two possibilities:

1) BMP4-ß-gal-expressing epithelial cells are taste bud stem cells. In general, stem cells in adult tissues divide very infrequently, occupy protected microenvironments or niches, and are predominantly in the resting phase (G0) of the cell cycle [72,73]. Cells in G0 typically do not express gene products of cell proliferation, and our failure to detect mitotic BMP4-ß-gal cells is consistent with this criterion. In skin, quiescent stem cells are normally intermingled with the transit amplifying population (see review, Alonso and Fuchs, 2003) [74]; similarly, BMP4-ß-gal cells are located in the basal epithelium surrounding taste buds, where we find that the majority of cells are actively cycling. Finally, Okubo [52] have recently suggested that multipotent stem cells located in the basal epithelium of fungiform papillae give rise to transit-amplifying daughters, which contribute to both taste buds and keratinocytes of the gustatory papillae. However, these latter studies involve genetic mapping of cells that express K14, while the precise identity of the initially labeled cells was not assessed. In our study, while some BMP4 cells express K14, another subset of perigemmal BMP4-ß-gal cells are not immunoreactive for K14, yet are located in the taste papilla epithelium immediately adjacent to taste buds. This heterogeneity in basal keratinocyte marker expression may reflect different functions for these cell subpopulations. Regardless of K14-immunoreactivity, however, we failed to detect proliferation of BMP4-positive cells.

2) A second possibility is that, instead of a taste bud stem cell population, BMP4-ß-gal-expressing epithelial cells represent a signaling center or compartment of the stem cell niche, which controls stem cell and/or transit amplifying cell division and/or differentiation. Our double-staining experiments for BMP4-ß-gal and proliferation markers revealed that the BMP4-expressing cells in the subepithelial lamina propria are also mitotically inactive, suggesting that in concert, these epithelial and mesenchymal BMP4 cells adjacent to taste buds may maintain the niche, or signaling center for taste bud stem cells. Interestingly, Miura [32] report that *Shh *is expressed exclusively in basal cells within taste buds, whereas *Ptch1*, a Shh receptor (reviewed by Ingham and McMahon, 2001) [75], is expressed in the epithelial cells outside of taste buds, but adjacent to intragemmal *Shh *expressing cells. Moreover, mitotic cells, as detected via short term BrdU incorporation are mainly in the *Ptch1 *expressing region, raising the likelihood that *Ptch1 *expressing cells comprise the transit amplifying population, and possibly the taste bud stem cells [33]. In both fungiform and circumvallate papillae, BMP4-ß-gal-expressing cells may lie adjacent to the *Ptch1 *expressing cells, or may express *Ptc1 *suggesting the possibility that BMP4 and SHH may coordinate taste cell turnover, as has been demonstrated for regeneration of a number of other epithelial appendages, including hair follicles [76,77].

## Conclusions

Our expression and birthdating data suggest both shared and divergent functions for BMP4 in adult mouse taste buds of the fungiform and cirumvallate papillae. In both papilla types, BMP4 is found in epithelial cells adjacent to taste buds proper, and these cells, while resident in the proliferative zone, are themselves, mitotically quiescent. This suggests that BMP4 may be involved in taste cell renewal for taste buds of both anterior and posterior taste papillae. However, only in the circumvallate taste buds, we find BMP4 expressed by taste cells within taste buds, and expression occurs in a subset of all taste cell types, with the exception of bitter detecting type II cells. Combined with our birthdating analysis, we suggest that BMP4 expression occurs in early differentiating taste cells, and thus may function in taste cell maturation; this putative maturation function would, however, be restricted to circumvallate taste buds.

## Methods

### Animals

BMP4^LacZ/+ ^mice

Tongues were obtained from BMP4^LacZ/+ ^mice [44] (line provided by Brigid Hogan), in which the BMP4 coding sequence has been replaced with a LacZ reporter sequence with a nuclear localization signal peptide, resulting in mice that accurately express nuclear ß-galactosidase in cells that express *BMP4 *[44,45,48]. For our in-house strain, these mice were bred onto a C57Bl6 background (10+ generations) from the original Sv129 background. BMP4-lacZ gene product is visualized directly with a histochemical X-gal reaction, or indirectly by immunostaining tissues with ß-galactosidase antiserum {see below). Mice were maintained and sacrificed in accordance with protocols approved by the Animal Care and Use Committee at the University of Colorado Denver, School of Medicine.

### Tissue preparation

Adult mice (2-6 months old) were anesthetized with 20% chloral hydrate, i.p. and perfused transcardially with 4% paraformaldehyde and heparin 10 U/L in 0.1M phosphate buffer. Tongues were dissected free from the lower jaw, and postfixed with 4% PFA overnight at 4°C (for immunostaining) or postfix omitted (for X-gal staining), followed by immersion in sucrose 20% in 0.1M PB overnight at 4°C (for immunostaining) or 2 hours (for X-gal staining). Cryoprotected tongues were embedded in OCT compound (Tissue Tek) and cryosectioned at 12 μm. Sections were thaw-mounted and stored at -20°C overnight before staining.

### Immunofluorescence for taste cells

For double immunostaining of ß-galatosidase (ß-gal) with taste cell immunomarkers, sections were washed in 0.1 M phosphate buffer for 10 minutes, permeabilized in 0.1% Triton X-100/PBS for 30 minutes, and blocked with blocking solution (0.2 M PB, 0.05 M NaCl, 0.1% triton X-100; 1% bovine serum albumin) with 5% normal goat serum for 2 hours at room temperature. Sections were then incubated overnight at 4°C in primary antisera diluted in blocking solution. Two primary antisera were applied to each slide: Guinea pig anti-ß-gal (1:1000) [53,78] and one of following: (1) rabbit anti-NTPDase2 (1:1000) [3]; (2) rabbit anti-Gustducin (1:1000; Santa Cruz); (3) rabbit anti-PLCß2 (1:1000; Santa Cruz); (4) rabbit anti-PGP 9.5 (1:1000; Chemicon); (5) rabbit anti-NCAM (1:1000; Chemicon); rabbit anti-serotonin (1:1000, ImmunoStar); or (7) rabbit anti-Sox 2 (1:1000; Chemicon). BMP4LacZ+/- mice used for serotonin immunocytochemistry were injected i.p. with 5-hydroxytryptophan (5-HTP; 80 mg/kg) 1 hour before perfusion [79]. After incubation with primary antisera overnight at 4°C, sections were washed with PBS for 90 minutes, and incubated with a mixture of fluorescently labeled secondary antisera in blocking solution for 2 hours at room temperature. Secondary antisera used were goat anti-guinea pig Alexa Fluor 488 (1:500), and goat anti-Rabbit Alexa Fluor 546 (1:1000) (Invitrogen). Sections were washed in 0.1 M PBS for 30 minutes and 0.1 M PB for 1 hour, counterstained with Hoecsht 33342 (Invitrogen), mounted in Fluoromount G and coverslipped for analysis using fluorescence and confocal microscopy.

### Immunofluorescence for cell cycle markers

For taste cell birthdating studies, BMP4^LacZ/+ ^adult mice were injected with 5-bromo-2-deoxyuridine (BrdU; Sigma; 100 mg/kg) between 10 am - 12 pm, twice with a 1-hour interval. No mice injected with BrdU died before being killed for the experiments. Mice were euthanized 6, 12, 18, 24, 48, or 72 hours after the first injection.

Sections for double immunofluorescent labeling of anti-BrdU and anti-ß-gal were first treated with methanol containing 3% hydrogen peroxide to block endogenous peroxidases, and then incubated in trypsin 0.05% at 37°C for 5 minutes. To detect incorporated BrdU, sections were pretreated with 4N HCl for 15 min at 50°C to denature DNA, washed thoroughly in 0.1 M PBS for 10 minutes, and incubated with 5% goat serum in blocking solution at room temperature for 2 hours, then treated with an avidin/biotin blocking kit (Vector Laboratories). Sections were incubated overnight at 4°C with mouse anti-BrdU biotin-conjugated antibody (1:1000; Zymed) and rabbit polyclonal antiserum against ß-gal (1:500; MP Biomedicals), washed with 0.1 M PBS for 2 hours, followed by incubation with goat anti-rabbit Alexa Fluor 488 (1:500), and Streptavidin conjugated with Alexa Fluor 546 (1:1000; Molecular Probes).

Alternatively, BrdU and BMP4-ß-gal double labeling was assessed using markers visible in bright field. For these experiments, sections from BrdU injected BMP4^LacZ/+ ^animals were first stained via X-gal histochemistry, then processed through methanol containing 3% hydrogen peroxide, trypsin 0.05% at 37°C, 4N HCl, and Avidin/Biotin blocking solutions as described above. Sections were then incubated with M.O.M. mouse Ig blocking reagent (Vector Laboratories) for 1 hour, then incubated overnight at 4°C with mouse anti-BrdU (1:500; G3G4, Developmental Studies Hybridoma Bank). After washing with PBS, sections were incubated for 30 minutes with biotin-conjugated anti-mouse IgG (Vector Laboratories), washed for 1 hour in PBS, and then treated with ABC solution (Vector Laboratories) for 90 min at room temperature. Finally sections were visualized with nickel-intensified DAB (Vector Laboratories) in peroxide substrate buffer for 3-5 minutes.

To detect PCNA and phospho-histone 3 (pH3) immunofluorescent cells, sections were bathed in sodium citrate buffer (pH 6.0) at 95°C for 15 minutes, then cooled to room temperature for 30 minutes. After washing thoroughly with 0.1 M PBS, sections were treated with blocking solution containing 5% normal goat serum for 2 hours, and then incubated with mouse anti-proliferating cell nuclear antigen (PCNA, Sigma) and rabbit anti-pH3 (Upstate Cell Signaling Solutions), both at 1:1000, overnight at 4°C. Secondary antisera were goat anti-rabbit Alexa Fluor 488 (1:1000), and goat anti-mouse Alexa Fluor 546 (1:1000; Molecular Probes).

For co-labeling of ß-galactosidase activity and Ki-67 or cytokeratin 14 (K14), animals were perfused with 4% paraformaldehyde in 0.1 M PB, then, without post-fixation, tongues were put in 20% sucrose for 2 hours at 4°C, embedded and cryosectioned at 12μm. Sections were post-fixed on slides for 5 minutes in 4% paraformaldehyde, rinsed twice in 0.1 M PBS, and reacted histochemically in X-gal solution (Chemicon) overnight at 4°C, then fixed with 4% paraformaldehyde in 0.1 M PB for 30 minutes at room temperature. After three washes in PBS, the sections were treated with 3% hydrogen peroxide, bathed in sodium citrate buffer (pH 6.0) at 95°C for 15 minutes, and then cooled to room temperature for 30 minutes. The sections were washed in PBS and subsequently incubated in blocking solution with 5% normal goat serum for 2 hours at room temperature; then avidin/biotin blocking kit (Vector Laboratories) was applied to the sections. The sections were then incubated overnight at 4°C with rabbit anti-Ki-67 antibody (1:200; Thermo Scientific) or guinea pig anti-K14 (gift from Dennis Roop, UC Denver School of Medicine). After washing in PBS for 3 hours, the sections were incubated for 1 hour at room temperature with biotin-conjugated anti-rabbit IgG (Vector Laboratories) or anti-guinea pig IgG (Vector Laboratories), diluted 1:500 in PBS with 0.1% Tween 20 and 2.5% normal goat serum. The sections were washed with PBS for 1 hour, and ABC solution (Vector Laboratories) was applied for 90 min at room temperature. After washing in PBS for 1 hour, sections were visualized with nickel-intensified DAB (Vector Laboratories) in peroxide substrate buffer for 5-10 minutes. Reacted slides were dehydrated with ethanol 50%, 70%, 95% and 100%, then cleared with xylene and coverslipped with Permount (Fisher Scientific).

### Analysis

For double-labeled immunofluorescent tissue, confocal images were obtained on an Olympus BX50 laser scanning confocal microscope. Images consisting of projected Z series of 0.75 μm optical sections were processed with Fluoview v5.0 software.

Double labeled cells were tallied via examination of staining in each optical section through a Z-series, and according to the following criteria dictated by the subcellular localization of a given marker: (i) overlap of nuclear staining (BrdU, Sox2) with BMP4-driven nuclear ß-galactosidase; (ii) cytoplasmic staining completely surrounding a ß-galactosidase positive nucleus (gustducin, PLCß2, PGP9.5, serotonin; e.g. Figure 3B',3C', 3D', 3E'); (iii) nuclear ß-galactosidase staining surrounded by at least 70% of the immunostained membrane profile of the same cell (NTPdase2, NCAM; e.g. Figure 3A',3F').

For tissue processed for X-gal with Ki-67 immunocytochemical staining, double labeling was assessed on a Zeiss Axioplan 2 microscope equipped with an Axiovision imaging system. Double labeling was defined by the expression of X-gal (blue) and Ki-67 in the same nucleus (e.g. Figure 6).

To assess the distribution of BMP4-ß-gal intragemmal cells in the taste buds of the circumvallate papilla, we analyzed 2 sections in the middle of each circumvallate papilla, divided the sections into left and right trenches, and then each trench into six regions: for the left trench -- upper lateral, middle lateral, bottom lateral, bottom medial, middle medial, and upper medial, and for the right, the mirror regions were assigned. We then counted the number of BMP4-ß-gal expressing cell profiles inside taste buds, but only counted positive cells in taste bud profiles which had both an apical pore and had a width between 35.1-46.1μm (equivalent to 1 standard deviation from the mean taste bud diameter obtained from measurements of 100 randomly selected taste buds from 3 mice = 40.6 +/-5.5μm). At least 50 taste buds for each circumvallate subregion were counted from 6 mice. Nomarski imaging was used to determine the borders of taste buds.

To evaluate the number of BrdU intragemmal cells at 24, 48 and 72 hours post-injection, 3 mice for each time point were used and at each post injection time point, we counted the number of BrdU-IR cells inside taste buds as defined above.

Digital images acquired via confocal or conventional fluorescence microscopy were converted to tiffs and contrast and brightness adjusted, cropped and assembled into multipanel plates using Adobe Photoshop.

The data for this study came from a total 42 BMP4-lacZ mice (age 2 to 6 months), with 3 mice for each marker or combination of markers e.g., Sox2, K14, cell cycle markers, or time points post-injection for the BrdU studies. All evaluations were based on counts from taste bud profiles with taste pores such that approximately 150 taste buds for circumvallate papillae and 100 taste buds for fungiform papillae were counted in each of 3 mice for each experimental staining permutation. All counting numbers were corrected by the Abercrombie correction factor [80].

## Authors' contributions

HMN carried out immunocytochemistry, histology and analysis. LAB was instrumental in the conceptual design, supervised experiments and data analysis. HMN and LAB wrote the manuscript. Both authors read and approved the final manuscript.
